# Pontine cavernous malformation: microsurgery evading the floor of the fourth ventricle

**DOI:** 10.3171/2019.7.FocusVid.19186

**Published:** 2019-07-01

**Authors:** Abdullah Keleş, Mehmet Volkan Harput, Uğur Türe

**Affiliations:** Department of Neurosurgery, Yeditepe University School of Medicine, Istanbul, Turkey

**Keywords:** brainstem surgery, cavernous malformation, cottonoid-guided intraoperative ultrasonography, floor of fourth ventricle, video

## Abstract

This video demonstrates resection of a left pontine cavernous malformation that is abutting the floor of the fourth ventricle (f4V). Even though accessing the lesion through the f4V seems to be reasonable, we used a lateral supracerebellar approach through the middle cerebellar peduncle to preserve especially the abducens and facial nuclei. After total resection the patient was neurologically intact at the 3-month follow-up. Postoperative MRI revealed 3.5-mm pontine tissue between the cavity and f4V that appeared to be absent in preoperative MRI. Approaching pontine lesions through the f4V is not the first choice. In our opinion, the philosophy of safe entry zones is a concept to be reassessed.

The video can be found here: https://youtu.be/1Jh6giZc-48.

## Transcript

In this video, we demonstrate the microneurosurgical removal of a pontine cavernous malformation through a left-sided retrosigmoid lateral supracerebellar approach.

A 35-year-old male came to our institution with a 2-month history of right-sided hemihypesthesia and right facial numbness.

The patient’s preoperative T2, T1 precontrast, and T1 postcontrast axial MR images revealed a multicystic hemorrhagic pontine cavernous malformation on the left side.

In this case, the lesion seemed to be almost on the floor of the fourth ventricle, and this might give the idea of approaching through the floor of the fourth ventricle.

However, in our opinion this route should not be the first choice. We considered an alternative approach because on the floor of the fourth ventricle abducens and facial nuclei were in very close proximity to the lesion.

This illustration summarizes the complex architecture of the floor of the fourth ventricle and demonstrates the relation of the lesion with the major vital neural structures, such as abducens and facial nuclei (Cavalcanti et al., 2019; Naidich et al., 2009; Nieuwenhuys et al., 2008; Párraga et al., 2016; Yaşargil, 1988).

Preoperative fiber tractography showed the medial lemniscus in blue color, which was compressed and displaced posteriorly, and the corticospinal tract in red color in relation to the lesion.

In this case, the brainstem entry point is decided with measurements made preoperatively on 1-mm-thick 3-Tesla 3D T1 turbo fast echo coronal, axial, and sagittal MR images. We decided to enter the brainstem through the middle cerebellar peduncle, approximately 3–4 mm superior and posterior to the trigeminal nerve root and 7–8 mm inferior to the tentorium.

In all brainstem cases we routinely use neuromonitoring.

With the patient in lateral position, lateral suboccipital retrosigmoid craniotomy is done on the left side.

The sigmoid and transverse sinuses are exposed.

Under the operating microscope, the dura is opened parallel to the transverse and sigmoid sinuses.

At the inferior aspect of the craniotomy, releasing CSF from the lateral cerebellomedullary cistern relaxes the posterior fossa.

The arachnoid membrane attached to the vasculature and neural tissue is released with sharp dissection.

The left-sided seventh and eighth nerve complexes, superior petrosal vein, and trigeminal nerve are recognized and released from the arachnoid membrane.

The superior cerebellar artery and trochlear nerve are visualized.

Then the superior cerebellar artery is mobilized.

Arachnoid dissection further relaxes the cerebellum and protects the cranial nerves and vessels from tension during the operation.

Between the fifth nerve and tentorium, the middle cerebellar peduncle is visualized.

A small cottonoid patty is placed over the middle cerebellar peduncle between trigeminal nerve and tentorium. And intraoperative real-time ultrasonography is performed from cranial to caudal to confirm the angle of the route.

The multicystic cavernous malformation and cottonoid patty are visualized with intraoperative ultrasonography. This is how we determined the best entry point.

In our experiences, this technique is more accurate than navigation system.

With intraoperative ultrasonography guided by the cottonoid patty, the most direct route to the lesion is delineated.

After we confirm the entry point, through the middle cerebellar peduncle a small incision of about 2 to 3 mm is made with a microblade parallel to the transverse pontine fibers.

With the tip of the bipolar forceps, the incision is gently widened.

Blunt dissection is done with suction and bipolar forceps to deepen the incision.

The cavity of the hematoma is opened, and its hemorrhagic content is evacuated.

The gliotic plane and cavernous malformation are identified.

Dissection is continued with a pressure-controlled suction and tip of bipolar forceps in low-power setting.

The cavernous malformation is dissected in circumferential fashion.

When the last attachment of the lesion is cut with bipolar forceps, the lesion is sucked by the suction mistakenly.

Therefore, unfortunately no sample could be collected for histopathological diagnosis.

For hemostasis, the use of bipolar forceps on a low-power setting is limited.

Exploring the surgical cavity with a high-definition neuroendoscope reveals no bleeding or residual lesion.

Through the middle cerebellar peduncle, a 5-mm incision is large enough to achieve good exposure and total resection with the help of cottonoid-guided intraoperative ultrasonography.

During the early postoperative period, the patient had transient diplopia, which completely resolved before he was discharged. He also had a mild balance problem and mildly increased right-sided hemihypesthesia. These deficits completely resolved by the 3-month follow-up.

Postoperative axial, coronal, and sagittal MR images showed total resection of the cavernous malformation as well as the accompanying venous anomaly which was preserved.

Our entry point through the left middle cerebellar peduncle is clearly seen.

In postoperative MR images one can easily recognize the 3.5-mm pontine tissue between the lesion cavity and the floor of the fourth ventricle. This tissue was appeared to be absent in the preoperative imaging due to the compression by the lesion.

Approaching the cavernous malformation through this 3.5-mm-thick tissue would have caused unwanted complications.

Even if one can manage to find that safe entry zone despite the anatomy being distorted by mass effect, that entry point will be widened during surgical manipulation, and will eventually harm the crucial neighboring neural tissue.

Approaching such lesions through the floor of the fourth ventricle should not be the first choice, and alternative approaches should be considered even when the lesion abuts the fourth ventricle.

Of course, when the lesion protrudes through the floor of the fourth ventricle, a direct posterior approach can be considered.

In our opinion, safe entry zones over the floor of the fourth ventricle are overexaggerated. This is a concept to be reassessed.

## Time points

0:34 Presentation of the case
0:45 Preoperative MR images
2:58 Patient positioning and craniotomy
3:28 Arachnoid dissection
4:28 Intraoperative cottonoid-guided ultrasonography
5:04 Brainstem incision
5:59 Cavernous malformation removal
6:24 Neuroendoscopic exploration of the surgical cavity
7:08 Postoperative MR images.
